# Foot Measurements From Three‐Dimensional Scans: Elinvision 3DST Reliability

**DOI:** 10.1002/jfa2.70070

**Published:** 2025-07-30

**Authors:** Petra J. Jones, Alex V. Rowlands, Melanie J. Davies, David Webb, Clare L. Gillies, Alam Shah

**Affiliations:** ^1^ Leicester Diabetes Centre University Hospitals of Leicester Leicester General Hospital Leicester UK; ^2^ Diabetes Research Centre University of Leicester Leicester General Hospital Leicester UK; ^3^ NIHR Leicester Biomedical Research Centre University of Leicester Leicester UK; ^4^ Alliance for Research in Exercise, Nutrition and Activity (ARENA) UniSA Allied Health and Human Performance Division of Health Sciences University of South Australia Adelaide Australia; ^5^ Leicester Real World Evidence Unit Diabetes Research Centre Leicester General Hospital Leicester UK; ^6^ Leicester Opcare Prosthetic, Orthotic, and Posture and Mobility Services Leicester UK

**Keywords:** 3D scanner, accuracy, foot measurement, footwear, orthosis, reliability

## Abstract

**Background:**

3D foot scanners such as the Elinvision 3DST foot scanner potentially offer a faster, alternative method to traditional plaster casting to produce orthotics or therapeutic footwear.

**Objective(s):**

To assess the reliability of 3DST‐derived foot length, orthogonal ball width, heel width and ball girth. We also compared 3D scanner and manually derived measures.

**Study Design:**

Repeated measures design.

**Methods:**

Two independent raters carried out three scans each of the right foot of 20 healthy participants (10 female) aged 18 years or over (mean age 38 ± 11.4 years) using the 3DST scanner (software v1.6.21.833). Manual foot measurements were taken by an experienced rater using Ritz stick and tape measure.

**Results:**

Intraclass correlation coefficients were excellent for both inter‐rater reliability (0.99–1.00, 95% confidence interval (CI) 0.97–1.00) and intra‐rater reliability (Rater 1: 0.98–1.00, 95% CI 0.96–1.00; Rater 2: 0.97–1.00, 95% CI 0.94–1.00). Standard error of the mean ranged from 0.1 to 0.4 cm both for scanner and manual measurements. The mean absolute differences between the scanner and manual measurements were ≤ 0.4 cm for foot length, orthogonal ball width (0.2–0.3 cm), ball girth and heel width (0.3–0.4 cm) but larger for foot waist, short heel, ankle circumference and anatomical ball width (0.5–1.1 cm).

**Conclusions:**

The 3DST scanner has potential application for capture of basic foot dimensions in footwear fit research. However, larger differences relative to manual measures for other dimensions limits its potential in orthotics or therapeutic footwear production.

## 1 Introduction

Incorrectly fitting footwear may increase the risk of diabetes‐related ulceration [1] and the probability of falls [2]. Good footwear fit is therefore crucial for people with rheumatoid arthritis, neuropathy, diabetes, or those recovering from stroke or with other conditions affecting the foot. Appropriate footwear fit and design can influence plantar pressure and foot health [3], by capturing variability in foot morphology for personalised footwear [4]. Typically, over two‐thirds of older people (> 60 years old) have been found to be wearing incorrectly fitting footwear [5]. Similarly, a large proportion of people with diabetes (59%–82%) [6, 7], rheumatoid arthritis (68%) [8] or those recovering from stroke (47%) [9] also routinely wear incorrectly fitting footwear. Numerous national and international guidelines specify parameters for correctly fitting footwear including the International Working Group on Diabetic Foot [10] and the World Health Organisation guidelines on diagnosis and management of diabetes [11].

Commonly feet are measured manually using devices such as Brannock devices [6, 12, 13], Ritz sticks [14] or sliding callipers [15] to determine foot length and width. These manual devices have been around for more than a century and are widely used to assess footwear fit [16]. They have the advantage of being easy‐to‐use, relatively inexpensive and are familiar to those being measured. However, the use of manual methods to take half a dozen or more measurements, particularly if repeated more than once, can be slow and therefore costly. Equally, the alternative of using plaster of Paris to capture a patient's foot morphology in order to make a last for therapeutic footwear can be time consuming for both orthotists and patients [17].

A large number of devices have emerged that are capable of scanning patients' feet from which various foot dimensions may be extracted. A 3D foot scanner is a device that combines cameras and lasers to capture the dimensions and morphology of a person's foot. To utilise such devices in the manufacture of therapeutic footwear or in footwear fit assessment as part of research, it is critical to establish the reliability of each device.

The Elinvision 3D foot scanner and earlier versions of its software have been used to scan the feet of nine runners [18], to determine factors affecting foot shape variation in 62 healthy volunteers [19] and to assess the effect of diabetes and neuropathy upon foot shape in 11 people with type 2 diabetes and 47 without [20]. However, these studies made use of older Elinvision FootIn3D [19] or Tiger [21, 22] scanners and not the more recent Elinvision 3DST model. Whilst the reliability of the Elinvision FootIn3D (an earlier version of the 3DST) was assessed by Schuster et al. [23] by scanning the left foot of 17 healthy subjects, this was based on measurement of mean and root mean square (RMS) Euclidean surface to surface distances. These metrics are less intuitive for orthotists, podiatrists or researchers assessing 3D foot scanners for potential use in capturing individual foot dimensions for medical research, footwear fit assessment or therapeutic footwear manufacture in that the reliability and accuracy in measuring particular foot dimensions is not reported. Furthermore, while Schuster et al. had two testers performing scans under three loading conditions (full bodyweight, half bodyweight and minimal weight (seated)), variation in individual foot dimensions of repeated measures (reliability) was not assessed [23]. The reliability of the 3DST scanner therefore is, to the author's knowledge, yet to be evaluated.

In this study, our primary objective was to assess the inter‐rater and intra‐tater reliability (intraclass correlation coefficient) of the Elinvision 3DST foot scanner‐derived basic foot measurements of foot length, orthogonal ball width, ball girth and heel width obtained from repeated measurements (three scans each by two independent raters). Secondary aims were to determine agreement between 3D scanner‐obtained basic foot measurements and manually derived measures through Bland‐Altman plots. This is essential to making therapeutic footwear.

## 2 Material and Methods

Our methodology adopts the CRITIC (Consistent Reporting Three‐Dimensional Scanning) 3D scanning checklist proposed by Allan et al. [24].

### 2.1 Participants

A total of twenty volunteers aged 18 years or over without diabetes were recruited to this study including 10 males and 10 females. The mean age ± standard deviation (SD) of participants was 38 ± 11.4 years ranging from 22 to 57 years. Participants' height, weight, and body mass index (BMI) were 1.7 m ± 0.1 (154.0–181.0 m), 78.8 ± 16.8 kg (56.4–109.9 kg), 27.0 ± 5.4 kg·m^−2^ (18.5–39.4) respectively. Seventy‐five percent of volunteers were Caucasian, 15% Black, and 10% Asian. Given a significance level of 0.05 and power of 0.80, a sample size of 14 was required, assuming an intraclass correlation coefficient (ICC) [25] of 0.95 based on the mean average found in previous 3D foot scanner reliability studies [26, 27, 28, 29, 30, 31, 32, 33] (Supporting Information S1: Table 1), with three repetitions per subject plus a drop‐out rate of 10%. Individuals were excluded from participation if unable to ambulate independently without walking aids, had obvious foot deformities, a history of foot surgery, balance impairment, any health conditions affecting foot morphology or pain, injuries or medical conditions affecting the legs or lower back. The study protocol was registered on ClinicalTrials.gov (NCT05676619) and approved by the research ethics committee of the University of Leicester. All subjects provided written informed consent prior to participation.

### 2.2 3D Scanning Participants

**FIGURE 1:**
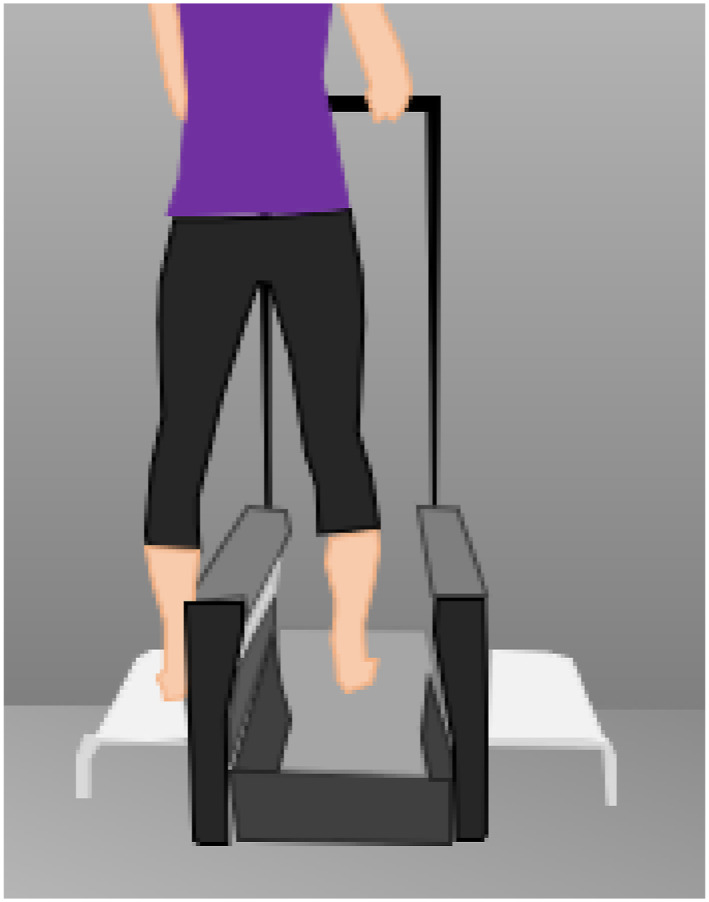
Half‐weight bearing position during 3D foot scan and floor marker for manual measurement.

Both bare feet were scanned using the Elinvision 3DST foot scanner (accuracy 0.5 mm, resolution: not known, capture duration: 5–15 s; reliability: not known; 1 RGB and 8 monochromatic cameras) and both feet manually measured, with right foot measurements analysed to assess reliability. During the scan, participants stood in a relaxed, half weight‐bearing position holding the handrail of the Elinvision 3D foot scanner (Figure 1). Two raters performed one set of three 3D foot scans each to assess intra‐ and inter‐rater reproducibility. The participant dismounted and remounted the scanner for each scan with both raters monitoring stance and posture, ensuring each participant was holding the handrail, looking straight ahead with feet facing forward. Neither rater accessed scanned foot measurements during the scanning process nor shared these with the other rater. Rater 1 was a researcher with only 6 months' prior experience of 3D foot scanning whereas Rater 2 was a trained orthotist based at Leicester General Hospital. For this reason, rater two performed all manual foot measurements using a Ritz stick to measure foot length, heel width, anatomical and orthogonal ball widths and a tape measure for all other measurements.

### 2.3 Experimental Setup Equipment

The Elinvision 3DST 3D foot scanner was used for all scans. Participants were asked to stand with one foot in the centre of the scanner side plate and the other in the centre of the scanner looking straight ahead gripping the handrail whilst scans were in progress (Figure 1). High resolution scans took 15 s to complete. The distance between the side plate and centre of the scanner was replicated using tape floor markers 33 cm apart to ensure participants' stances were identical during both scans and manual measurement.

Ritz sticks have been used to make foot measurements for over a century, including within research studies assessing footwear [14]. A Ritz stick was used to make foot measurements in centimetres (cm) for both manual foot length, orthogonal ball width and heel width whilst participants were standing. A tape measure was used to manually measure ball girth, foot waist, short heel and ankle circumference. All traditional anthropometry measurements were taken by an experienced orthotist (AS).

### 2.4 Foot Scanner Data Processing

**FIGURE 2:**
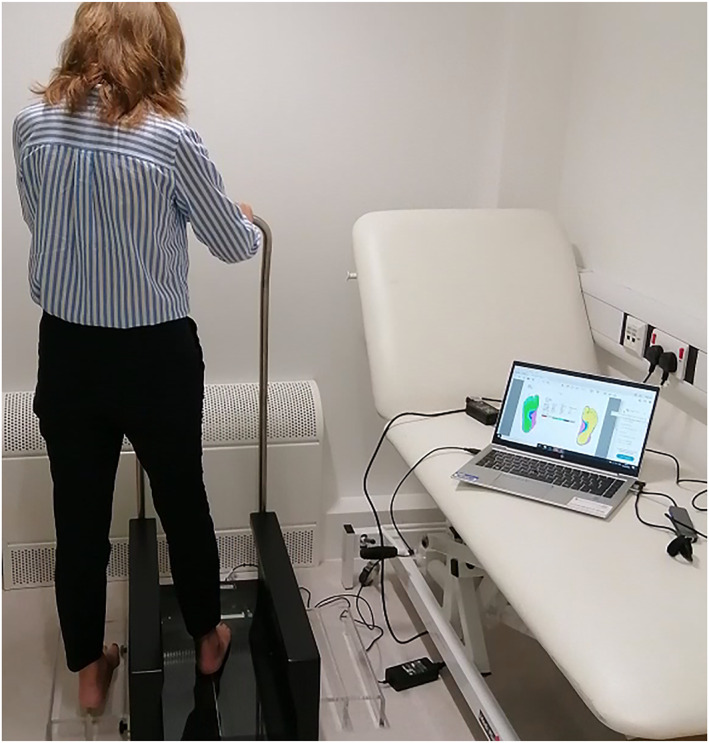
3DST foot scanner with Windows 10 Bang and Olufsen EliteBook Intel Core i5 laptop (8 GB RAM, CPU @ 1.60 GHz).

Foot lengths, anatomical ball width and short heel were extracted automatically by version 1.6.21.833 of Elinvision 3DST software installed on a windows laptop (Figure 2). Foot length was automatically measured by the software which draws a boundary box around scanned points lower than 10 cm, from which the measured length is derived.

**FIGURE 3:**
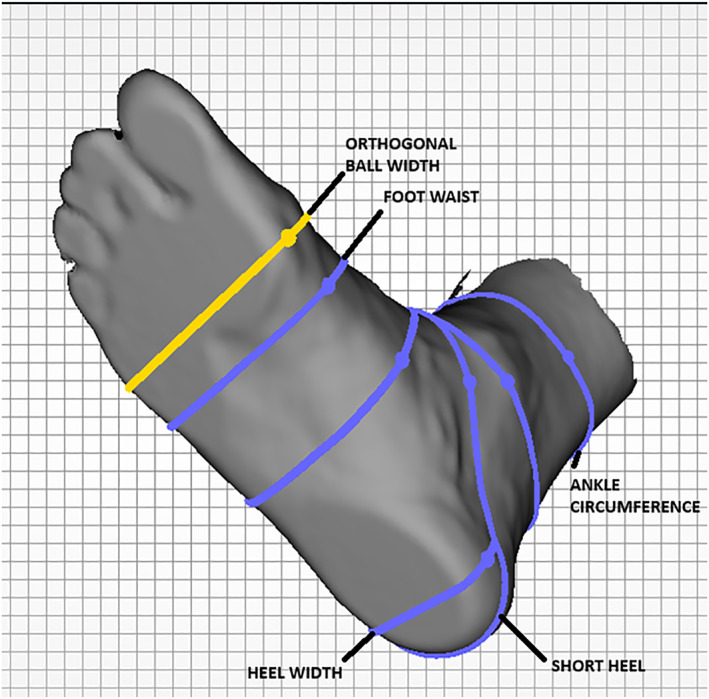
Dot marker positioning using Elinvision software.

All other measurements (orthogonal ball width, ball girth, foot waist, heel width, and ankle circumference) relied on manual re‐positioning of anatomical measurement markers within Elinvision software by one rater (PJ) after a period of several months of weekly training under orthotist supervision (AS) (Figure 3). An average of scan‐derived measurements was used for comparison with manual measurements (mean absolute difference).

### 2.5 Data Analysis

Primary outcomes included intra‐rater reliability based on intraclass correlation coefficient (ICC 3, k) [25] to assess variation in measurements by each rater and inter‐rater reliability based on the intraclass correlation coefficient (ICC 2,k) [25] to determine agreement between the two raters for the four primary outcome foot dimensions (foot length, orthogonal ball width, ball girth, heel width) essential in both therapeutic footwear manufacture and footwear fit research. Interpretation of ICC values was based on a prior 3D foot scanner reliability study where less than 0.5 ICC was considered poor, 0.5–0.75 as moderate, 0.75–0.90 as good, and above 0.90 as excellent [28]. Analysis was based on the scanned right foot as in previous studies [28, 29, 30] with left foot scan data provided in supplemental data (see Supporting Information S1: Figures S1 and S3) for comparison. Secondary outcomes were intra‐rater and inter‐rater reliability for four additional foot dimensions including anatomical ball width, foot waist, short heel, and ankle circumference with Bland–Altman plots [34] (see Supporting Information S1: Figure S2) and Standard error of the mean (SEM, see Supporting Information S1: Table 2) for all foot dimensions obtained through scanner or manual measurements. SEM was calculated by taking the standard deviation of these measurements and dividing these by the square root of the sample size. The mean absolute difference was calculated by obtaining the absolute difference of manual measurements subtracted from scanned measurements.

## 3 Results

### 3.1 Inter‐Rater Reliability

Intraclass correlation coefficient (ICC) for inter‐rater reliability for the Elinvision 3DST foot scanner ranged from 0.99 to 1.00 for primary outcome foot dimensions of foot length, orthogonal ball width, ball girth and heel width (Table 1). Degree of rater experience did not affect measurement reliability (Table 1). ICC for inter‐rater reliability for secondary outcome foot dimensions were also excellent and ranged from 0.96 to 1.00 for anatomical ball width, ankle circumference, foot waist and short heel (Table 1). The stated ball girth ICC excludes one obviously spurious value (< 50% of other scanned ball girth measurements) produced by the software.

**TABLE 1:** Inter‐rater reliability: intraclass correlation coefficient (2, k).

Foot dimension	ICC (95% confidence interval)
Primary outcomes	
Foot length	1.00 (1.00, 1.00)
Orthogonal ball width	0.99 (0.97, 1.00)
Ball girth*	0.99 (0.97, 1.00)
Heel width	0.99 (0.98, 1.00)
Secondary outcomes	
Foot waist	0.98 (0.94, 0.99)
Short heel	1.00 (1.00, 1.00)
Ankle circumference	1.00 (0.99, 1.00)
Anatomical ball width	0.96 (0.90, 0.99)

*Note:* All data is provided for the right foot. *Ball girth ICC calculated after excluding one spurious software value.

### 3.2 Intra‐Rater Reliability

Intra‐rater reliability was also excellent for both raters for all foot dimensions (Rater 1: 0.92–1.00; Rater 2: 0.97–1.00, Table 2).

**TABLE 2:** Intra‐rater reliability: intraclass correlation coefficient (3, k).

Foot dimension	Rater 1	Rater 2
	ICC (95% confidence interval)	ICC (95% confidence interval)
Primary outcomes		
Foot length	1.00 (1.00, 1.00)	1.00 (1.00, 1.00)
Orthogonal ball width	0.99 (0.97, 0.99)	0.99 (0.97, 0.99)
Ball girth*	0.99 (0.98, 1.00)	0.99 (0.98, 1.00)
Heel width	0.98 (0.96, 0.99)	0.97 (0.94, 0.99)
Secondary outcomes		
Foot waist	0.92 (0.84, 0.97)	0.97 (0.93, 0.99)
Short heel	1.00 (0.99, 1.00)	1.00 (1.00, 1.00)
Ankle circumference	0.99 (0.97, 1.00)	0.98 (0.96, 0.99)
Anatomic ball width	0.92 (0.84, 0.97)	0.97 (0.93, 0.99)

*Note:* All data is provided for the right foot. *Ball girth ICC calculated after excluding one spurious software error (< 50% other scanned ball girth values).

### 3.3 Bland‐Altman Plots

**FIGURE 4:**
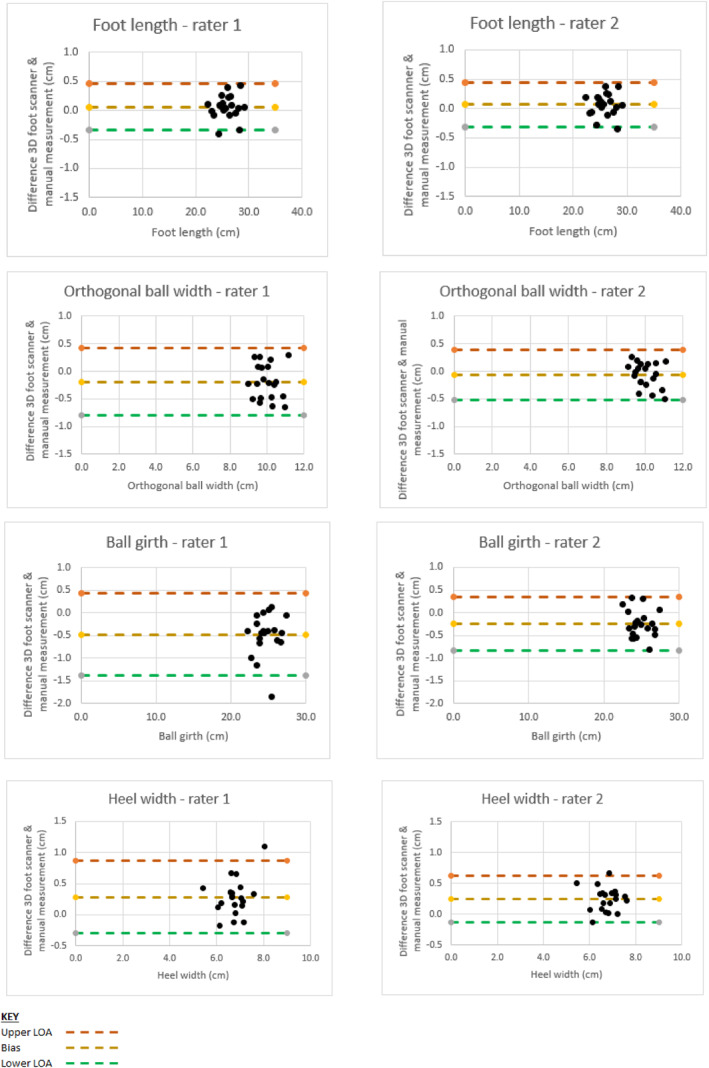
Primary outcome Bland‐Altman plots (right foot).On outlier values: Ball girth ‐rater 1

Figure 4 shows Bland–Altman Plots with 95% limits of agreement for foot length, orthogonal ball width, ball girth and heel width for each rater. The dashed central yellow line represents the mean difference between 3D foot scanner and manual measurements of foot dimensions (Ritz stick for foot length, orthogonal ball width, and heel width, and tape measure for ball girth) with upper and low 95% limits of agreement represented by orange and green dashed lines respectively.

### 3.4 Mean Absolute Difference

Mean absolute difference was calculated by obtaining the absolute difference of manual measurements subtracted from scanned measurements to provide a comparison between them. The mean absolute difference between 3D foot scanner‐derived measurements and manual measurements was deemed acceptable if within 0.4 cm given its equivalent to a half‐size in UK shoe sizes. A full shoe size difference has been used as a threshold for research studies assessing the effect of incorrectly fitting footwear upon ulceration risk in people with diabetes [35]. The mean absolute differences for core measurements (foot length, orthogonal ball width, heel width, ball girth) were less than or equal to 0.4 cm (see Table 3 below). Larger mean absolute differences between manually measured and 3D foot scanner values were found for other measurements (Table 3), particularly ankle circumference and anatomical ball width (1.0–1.1 cm). Further study data on mean absolute differences including comparison with other studies (Supporting Information S1: Table 4) and additional data for the left foot (Supporting Information S1: Table 5) are provided in the supplemental material.

**TABLE 3:** Mean absolute difference between manual and scanner foot measurements.

Foot dimension	Mean absolute difference (cm)
Foot length	0.2
Orthogonal ball width	0.3
Ball girth	0.4
Heel width	0.3
Foot waist	0.5
Short heel	0.6
Ankle circumference	1.0
Anatomical ball width	1.1

*Note:* All values shown are for the right foot in centimetres (cm).

### 3.5 Standard Error of the Mean

Standard error of the mean (SEM) of 3D foot scanner‐derived foot measurements varied from 0.1 cm (heel width) to 0.5 cm (short heel) (Table 4) for each rater. SEM values were almost identical for each rater using the 3D foot scanner. The SEM of manual measurements were very similar to SEM values using the 3D foot scanner (Supporting Information S1: Table 6 provides other studies' SEM data for comparison).

**TABLE 4:** Standard error of the mean (SEM) for 3D foot scanner and manual measurements.

	3D foot scanner		Manual measurement	
Foot dimension	Rater 1	Rater 2	Rater 2	Method used
Foot length	0.4	0.4	0.4	Ritz stick
Orthogonal ball width	0.1	0.1	0.1	Ritz stick
Ball girth	0.3	0.3	0.3	Tape measure
Heel width	0.1	0.1	0.1	Ritz stick
Foot waist	0.4	0.3	0.4	Tape measure
Short heel	0.5	0.5	0.5	Tape measure
Ankle circumference	0.4	0.4	0.5	Tape measure
Anatomic ball width	0.2	0.3	0.2	Ritz stick

*Note:* All values shown are in centimetres for the right foot.

## 4 Discussion

We found excellent inter‐rater and intra‐rater reliability and acceptable mean absolute differences as compared with manual measurements for the primary outcomes of foot length, ball width, heel width and ball girth. Our results suggest potential for application of the 3DST 3D foot scanner in research capturing these basic foot dimensions. We selected these dimensions given their use in both diabetes foot ulcer prevention, footwear fit research more generally and therapeutic footwear production. However, mean absolute differences relative to manual measures were larger for the secondary outcomes of anatomical ball width, foot waist, short heel, and ankle circumference limiting any potential use of Elinvision 3DST software (v1.6.21.833) in therapeutic footwear production, where these measurements are used in making custom lasts or selecting modular lasts. A limitation of our study concerns any errors which arise during manual measurements.

The inter‐rater reliability intraclass correlation coefficients (ICC) 3DST obtained foot length (1.00) appear better [28, 29], or comparable [26, 30, 36] with those in other scanner reliability studies (Supporting Information S1: Table 3). Inter‐rater reliability for orthogonal ball width, ball girth and heel width (all ICC 0.99) were either higher or close to values achieved by other scanners (Supporting Information S1: Table 3). The difference in rater experience did not significantly affect scanner inter‐rater reliability (0.99–1.00, Table 1) and standard error of the mean was almost identical (Table 4). Intra‐rater reliability ICC for foot length, orthogonal ball width, ball girth and heel width in our study (Rater 1: 0.98–1.00; Rater 2: 0.97–1.00) also exceeded that of an earlier study: (Rater 1: 0.76–0.99; Rater 2: 0.83–0.99) [36] (Supporting Information S1: Table 3). However, it must be noted that methodology in these studies varies: for example, number of participants (10 [27] to 130 [29]), raters (one rater [26, 27, 29, 30] or two raters [28] Supporting Information S1: Table 1) number of scans (typically two [28, 29, 30] or 3 [26, 27]), and the interval between scans (none [26], 1 week [30], or even 4 weeks [28]). The type of ICC used also varied in these studies: ICC (2,1) in Witana et al. [36], ICC (3,1) in Menz et al. [30] or is frequently unreported [26, 27, 28, 29].

When comparing mean absolute difference between 3DST‐derived foot measurements and manual measurements with a similar study [29], results are mixed. For example, we find mean absolute difference for foot length of 2 versus 1.6 mm; orthogonal ball width: 3 versus 11.9 mm; anatomical ball width: 10 versus 7 mm; heel width: 3 versus 0.6 mm. However, there were notable differences between studies: for example, the earlier study was much larger (*n* = 130) with only one rater making just two scans, with use of a digital calliper rather than Ritz stick and tape measure for manual measurements. Mean absolute difference was not assessed for ball girth, foot waist, short heel or ankle circumference [29]. Our study was, in fact, the only one among these studies [26, 27, 28, 29, 30, 36] to assess the mean absolute difference of either ankle circumference or foot waist measurements. Only one study (*n* = 20) reported mean absolute difference for short heel (< 1 mm) [36]. This study evaluated mean absolute difference for the YETI‐I scanner in 18 foot dimensions as compared with manual measurements using set square, tape measure and callipers. Reported mean absolute difference was < 3 mm for all length and width measurements, and < 1.1 mm for ball girth, foot waist and ankle circumference [36] based on two scans by two raters. Given the large mean absolute differences we found for anatomical ball width, foot waist, short heel and ankle circumference, we were unable to establish that accuracy was sufficient for therapeutic footwear manufacture using the 3DST scanner.

Strengths of our study include ensuring the same distance between feet during both scans and manual measurement using floor markers, and the use of both an experienced and less experienced rater to confirm the 3DST scanner might equally be used by both. Limitations of our study are its size of 20 participants where a larger study of 50 participants would be required to detect a difference from a clinically meaningful null hypothesis of ICC = 0.90 with ≥ 80% power. Our study was carried out exclusively with healthy participants for safety reasons given its' first ever use at our hospital and we acknowledge that a cohort with diabetes may present with deformities, amputations, neuropathy, rheumatoid arthritis, oedema and other foot pathologies. Further validation in such a cohort is therefore necessary prior to clinical implementation. We also note the absence of dot markers as a method for duplicating sites of 3D foot scanner and manual measurements. Our attempts using markers and protruding adhesive stickers as dot markers were unsuccessful. Every effort was made to ensure participants' stance, foot and handrail position remained uniform prior to scanning but we acknowledge the degree of relaxation among participants may have fluctuated. We acknowledge that our assessment of mean absolute difference relies on manual foot measurements, which themselves may also be subject to human error. To minimise this, measures were taken by an experienced trained orthotist. Equally, manual positioning of anatomical markers by one rater to extract orthogonal ball width, ball girth, foot waist, heel width and ankle circumference may have affected mean absolute difference and ICCs. Sample size is limited to 20 participants and therefore representativeness may be another limitation as with all assessments of 3D foot scanner reliability.

Regarding the use of 0.4 cm as a target for mean absolute difference, we note that in one study [29], a more rigorous standard of 0.2 cm was used as the maximum allowable error between the scanner extracted value and manual measurements, citing the International Standard (ISO20685) for 3‐D scanning methodologies for internationally compatible anthropometric databases [37]. We chose 4 mm given the potential human error that might limit our study's precision in assessing mean absolute difference. Error margins for human measurements using callipers [26] or tape measures [38] have been estimated to be 0.1–0.2 cm. An additional analysis using the ISO threshold of 0.2 cm would likely necessitate comparison with a validated 3D foot scanner of known accuracy. Only the mean absolute difference of foot lengths were within the 0.2 cm threshold although both heel width and orthogonal ball width were close (0.3 cm) although these values are subject to the aforementioned potential human error in manual measurement.

We did encounter some challenges with our experimental setup (version 1.6.21.833 of Elinvision scanner software, Windows EliteBook laptop) which included sporadic difficulties with saving edited scans after manually repositioning anatomical markers. To avoid data loss, a workaround was found of re‐saving if saved file sizes were found to be low, implying incomplete data saved.

There were also some instances where despite obtaining good scans, certain dimensions had zero values within the software: for instance, with regard to orthogonal ball width, three of the 120 right foot (2.5%) and 1 of the 120 (0.8%) left foot orthogonal ball width measurements were affected (prior to any edits or repositioning of anatomical markers). Similar numbers of ball girth and heel width values were affected. These missing values were excluded from our analysis. Foot length values were not affected. This would mean carrying out suitable checks (running the report containing foot measurements to look for any possible errors, that might merit carrying out a re‐scan) if the device were used in practice.

The 3DST scanner can export the subject's left or right plantar footprint with colour codes indicating how far away in millimetres various areas of the plantar surface are away from the scanner bed. This could potentially be a useful feature for personalised orthotic, insole or footwear fitting. However, evaluation of its accuracy or reliability fell outside the scope of this study and so it is unclear how it might integrate with either footprint or pedobarograph‐based plantar pressure assessments to enhance footwear customisation.

## 5 Conclusion

The Elinvision 3DST 3D foot scanner has excellent inter‐rater and intra‐rater reliability for foot length, orthogonal ball width, ball girth and heel width, comparable with other 3D foot scanners evaluated in similar studies. Mean absolute differences between manual (Ritz stick or tape measure) and scanner measurements were acceptable for these dimensions (0.2–0.4 cm) with potential application to capture these basic foot measurements useful in research contexts. However, circumferential measures including short heel or ankle circumference were 0.6–1.0 cm which limits potential use of the scanner using software version 1.6.21.833 in therapeutic footwear production.

## Author Contributions

**Petra J. Jones:** conceptualization, data analysis, formal analysis, funding acquisition, investigation, methodology, project administration, resources, software, supervision, validation, visualisation, writing – original and draft, writing – review and editing. **Alex V. Rowlands:** writing – review and editing. **Melanie J. Davies:** writing – review and editing. **David Webb:** project administration, writing – review and editing. **Clare L. Gillies:** formal analysis, supervision, writing – review and editing. **Alam Shah:** writing – review and editing.

## Conflicts of Interest

The authors declare no conflicts of interest.

## Supporting information

Supporting Information S1

## Data Availability

The data that support the findings of this study are available from the corresponding author upon reasonable request.
