# Supplementary material for: Foot Measurements From Three‐Dimensional Scans: Elinvision 3DST Reliability
Source: J Foot Ankle Res. 2025 Jul 30;18(3):e70070. doi: 10.1002/jfa2.70070 (PMC12309729; doi:10.1002/jfa2.70070)
Supplement: Supplementary file 1 — Supporting Information S1 [file JFA2-18-e70070-s001.docx]

**Supplemental Materials
FIG.S1 Primary outcome Bland Altman Plots (left foot)**
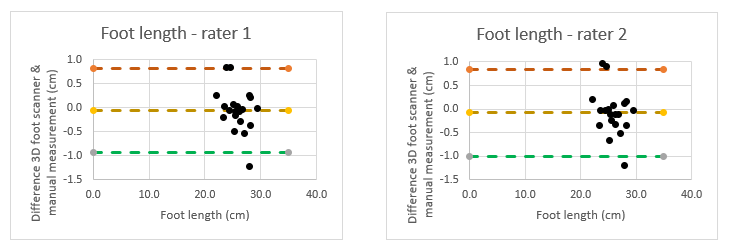


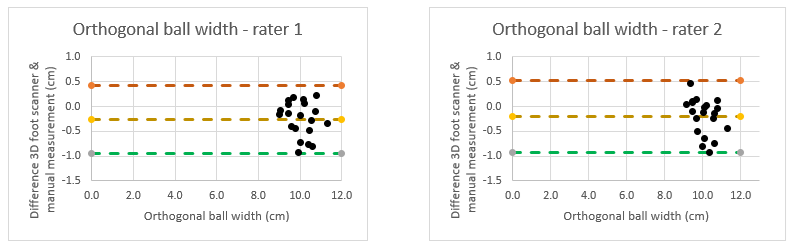

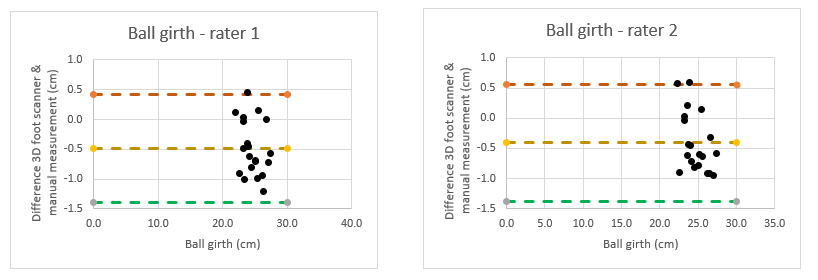


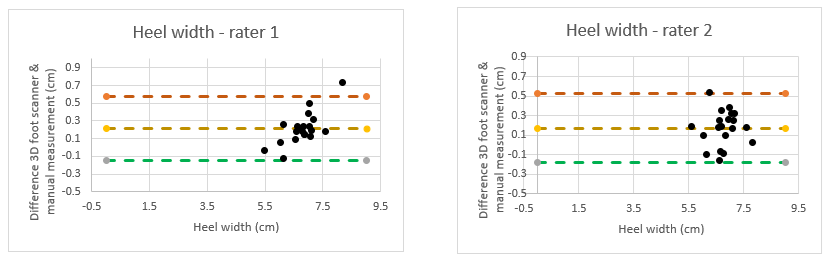


**FIG.S2 Secondary outcome Bland Altman Plots (right foot)**
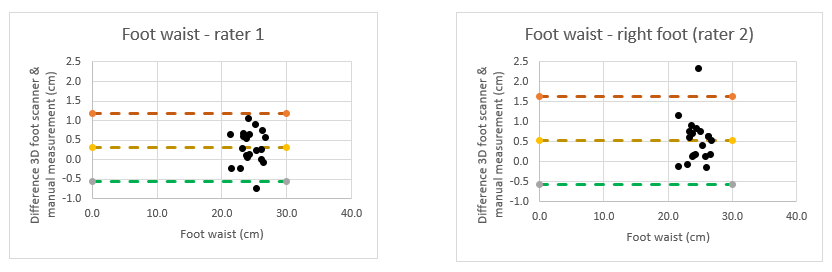
 **
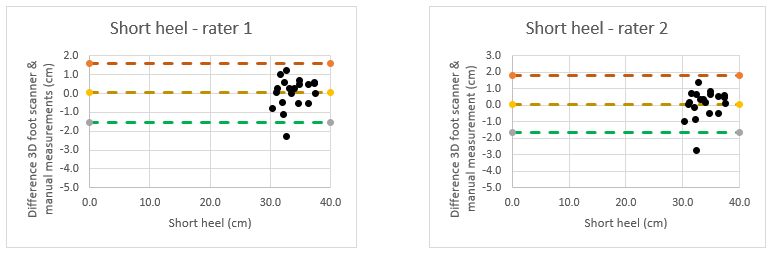


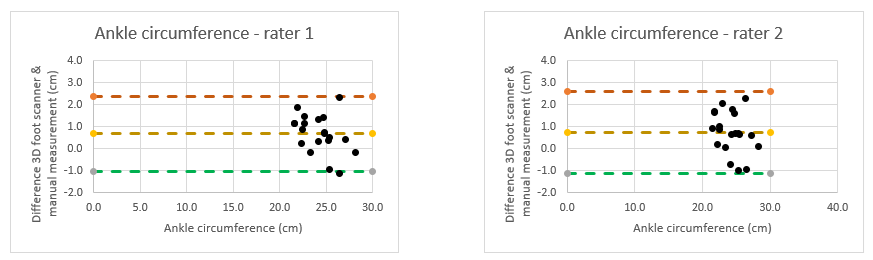
**

**
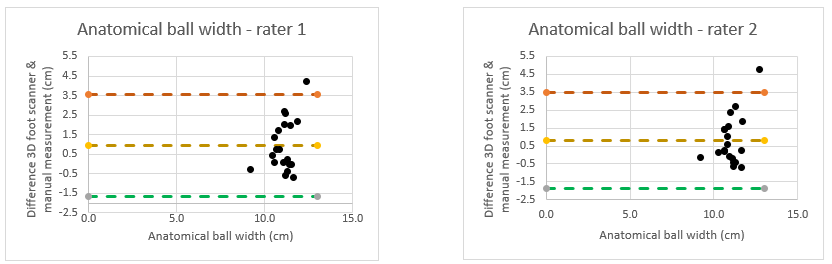
**

**FIG.S3 Secondary outcome Bland Altman Plots (left foot)


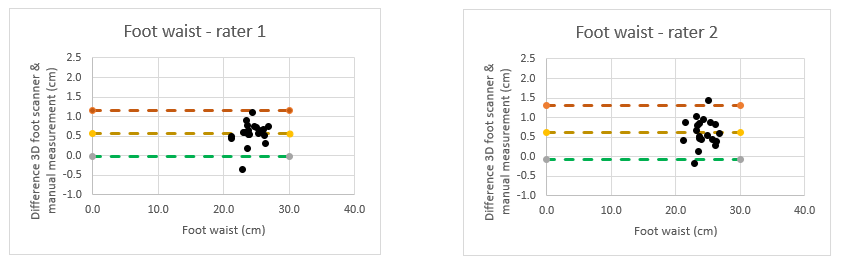
**


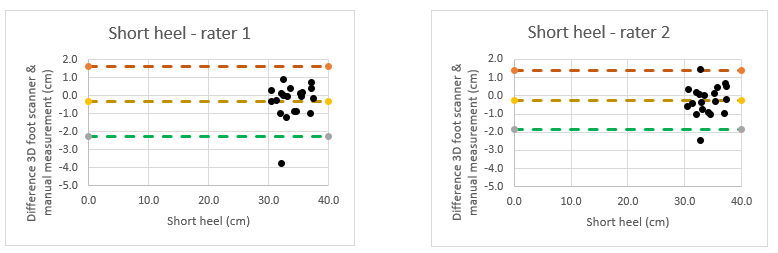


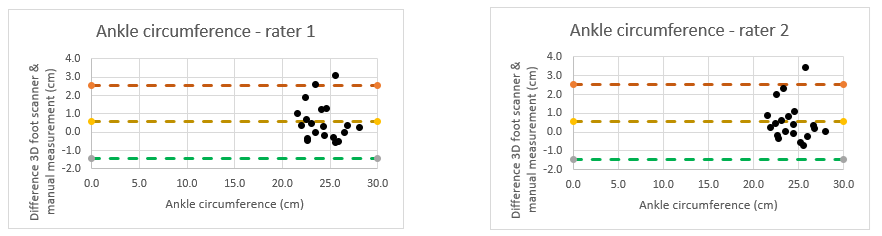


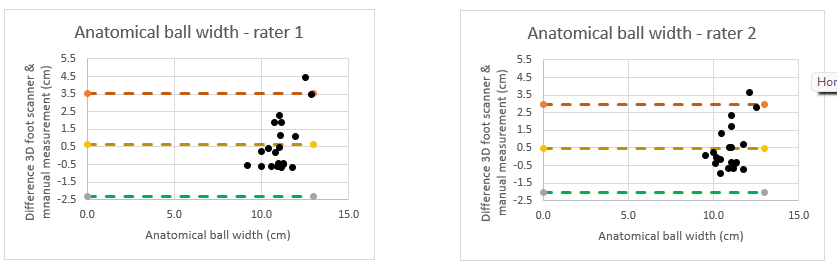


**SUPP. TABLE 1 3D foot scanner reliability: Intraclass Correlation Coefficient ranges**

|  |  | **Intraclass Correlation Coefficient (95% CI)** | | | |
| --- | --- | --- | --- | --- | --- |
| **Lead author** | **Study  size** | **Foot Length (FL)** | **Orthogonal ball width (OBW)** | **Heel width (HW)** | **Ball girth (BG)** |
| Ballester [26] | 16 | 0.999 | 0.992 | -- | 0.994 |
| De Mits [27] | 10 | 0.997 L 0.998 R | 0.990 L 0.991 R | 0.996 L 0.997 R | 0.995 L 0.996 R |
| Hassan [28] | 30 | 0.99 (0.99-1.00) | 0.98  (0.96-0.99) | 0.91 (0.81-0.95) | 0.99 (0.97-0.99) |
| Lee [29] | 130 | 0.98 | 0.96 | 0.96 | -- |
| Menz [30] | 20 | 0.999 (0.996-0.999) | 0.968 (0.920-0.987) | -- | 0.964 (0.910-0.985) |
| Powers [31] | 30 | 0.99 (0.99-1.00) | 0.98 (0.95-0.99) | 0.98  (0.95-0.99) | -- |
| Shariff [32] | 223 | -- | -- | -- | -- |
| Telfer [33] | 22 | 0.94 (0.84-0.98) T1 0.92 (0.81-0.96) T2 | 0.87 (0.72-0.95) T1 0.87 (0.74-0.95) T2 | -- | 0.96 (0.90-0.98) T1 0.95 (0.88-0.98) T2 |
| **Mean average** | | **0.98** | **0.95** | **0.97** | **0.98** |

**SUPP. TABLE 2 Standard Error of the Mean (SEM) for 3D foot scanner and manual measurements**

|  | **3D foot scanner** | | **Manual measurement** | |
| --- | --- | --- | --- | --- |
| **Foot dimension** | **Rater 1** | **Rater 2** | **Rater 2** | **Method used** |
| Foot length (FL) | 0.4 | 0.4 | 0.4 | Ritz stick |
| Orthogonal ball width (OBW) | 0.1 | 0.1 | 0.1 | Ritz stick |
| Ball girth (BG) | 0.3 | 0.3 | 0.3 | Tape measure |
| Heel width (HW) | 0.1 | 0.1 | 0.1 | Ritz stick |
| Foot waist (FW) | 0.4 | 0.3 | 0.4 | Tape measure |
| Short heel (SH) | 0.5 | 0.5 | 0.5 | Tape measure |
| Ankle circumference (AC) | 0.4 | 0.4 | 0.5 | Tape measure |
| Anatomic ball width (ABW) | 0.2 | 0.3 | 0.2 | Ritz stick |

All values shown are for the right foot in centimetres

**SUPP. TABLE 3 Comparison of 3DST scanner reliability (ICC) with other 3D foot scanner studies**

| **Inter-rater reliability – Intraclass Correlation Coefficient (ICC)** | | | | | | | |
| --- | --- | --- | --- | --- | --- | --- | --- |
| **Foot dimension** | **This study** | **Ballester [26]** | **De Mits [27]** | **Hassan [28]** | **Lee  [29]** | **Menz [30]** | **Witana [36]** |
| Foot length (FL) | 1.00 | 1.00 | 1.00 | 0.99 | 0.98 | 1.00 | 1.00 |
| Orthogonal ball width (OBW) | 0.99 | 1.00 | 0.99 | 0.98 | 0.96 | 0.97 | 0.99 |
| Ball girth (BG) | 0.99 | 1.00 | 1.00 | 0.99 | N/A | 0.96 | 1.00 |
| Heel width (HW) | 0.99 | N/A | 1.00 | 0.91 | 0.96 | N/A | 0.99 |
| Foot waist (FW) | 0.98 | N/A | N/A | N/A | N/A | N/A | 0.99 |
| Short heel (SH) | 1.00 | N/A | N/A | N/A | N/A | N/A | 0.98 |
| Ankle circumference (AC) | 1.00 | N/A | N/A | N/A | N/A | N/A | 0.99 |
| Anatomical ball width (ABW) | 0.96 | N/A | N/A | 0.98 | 0.95 | N/A | N/A |
| **Intra-rater reliability – Intraclass Correlation Coefficient (ICC)** | | | | | | | |
|  | **This study** | | **Hassan et al.  [28]** | | **Witana et al. [36]** | |  |
| **Foot dimension** | **Rater 1** | **Rater  2** | **Rater  1** | **Rater  2** | **Rater  1** | **Rater  2** |  |
| Foot length (FL) | 1.00 | 1.00 | 0.99 | 0.99 | 1.00 | 1.00 |  |
| Orthogonal ball width (OBW) | 0.99 | 0.99 | 0.97 | 0.98 | 0.99 | 0.98 |  |
| Ball girth (BG) | 0.99 | 0.99 | 0.98 | 0.98 | 0.99 | 0.99 |  |
| Heel width (HW) | 0.98 | 0.97 | 0.76 | 0.83 | 0.98 | 0.99 |  |
| Foot waist (FW) | 0.92 | 0.97 | N/A | N/A | 1.00 | 0.99 |  |
| Short heel (SH) | 1.00 | 1.00 | N/A | N/A | 0.93 | 1.00 |  |
| Ankle circumference (AC) | 0.99 | 0.98 | N/A | N/A | 0.99 | 0.99 |  |
| Anatomical ball width (ABW) | 0.92 | 0.97 | 0.99 | 0.98 | N/A | N/A |  |

N/A – Not applicable, foot dimension not assessed in study.

**SUPP.TABLE 4 Mean absolute difference in this 3DST scanner study (right foot) and other studies**

| **Mean absolute different (MAD)** | | | | | | | |
| --- | --- | --- | --- | --- | --- | --- | --- |
| **Foot dimension** | **This study (right foot)** | **Ballester [26]** | **De Mits [27]** | **Hassan [28]** | **Lee  [29]** | **Menz [30]** | **Witana [36]** |
| Foot length (FL) | 0.2 | N/A | N/A | N/A | 0.16 | N/A | 0.03-0.13 |
| Orthogonal ball width (OBW) | 0.3 | N/A | N/A | N/A | 1.19 | N/A | 0.09-0.13 |
| Ball girth (BG) | 0.4 | N/A | N/A | N/A | N/A | N/A | 0.36-0.43 |
| Heel width (HW) | 0.3 | N/A | N/A | N/A | 0.06 | N/A | N/A |
| Foot waist (FW) | 0.5 | N/A | N/A | N/A | N/A | N/A | N/A |
| Short heel (SH) | 0.6 | N/A | N/A | N/A | N/A | N/A | 0.03-0.08 |
| Ankle circumference (AC) | 1.0 | N/A | N/A | N/A | N/A | N/A | N/A |
| Anatomical ball width (ABW) | 1.1 | N/A | N/A | N/A | 0.43 | N/A | N/A |

All values shown are in centimetres.

**SUPP.TABLE 5 Mean absolute difference between manual and scanner foot measurements (left foot)**

| **Foot dimension** | **Mean absolute difference (cm)** |
| --- | --- |
| Foot length (FL) | 0.3 |
| Orthogonal ball width (OBW) | 0.3 |
| Ball girth (BG) | 0.6 |
| Heel width (HW) | 0.2 |
| Foot waist (FW) | 0.6 |
| Short heel (SH) | 0.6 |
| Ankle circumference (AC) | 0.8 |
| Anatomical ball width (ABW) | 1.0 |

All values shown are in centimetres for the left foot.

**SUPP. TABLE 6 Standard Error of the Mean in this 3DST scanner study (right foot) and other studies**

| **Standard Error of the Mean (SEM)** | | | | | | | | |
| --- | --- | --- | --- | --- | --- | --- | --- | --- |
| **Foot dimension** | **This study** | | **Ballester [26]** | **De Mits [27]** | **Hassan [28]** | **Lee  [29]** | **Menz [30]** | **Witana [36]** |
|  | **T1** | **T2** |  |  |  |  |  |  |
| Foot length (FL) | 0.4 | 0.4 | 0.6-1.0 | 0.03-0.27 | N/A | N/A | N/A | N/A |
| Orthogonal ball width (OBW) | 0.1 | 0.1 | 0.6-0.7 |  | N/A | N/A | N/A | N/A |
| Ball girth (BG) | 0.3 | 0.3 | 1.0-1.2 | 0.06-0.23 | N/A | N/A | N/A | N/A |
| Heel width (HW) | 0.1 | 0.1 | N/A | 0.03-0.27 | N/A | N/A | N/A | N/A |
| Foot waist (FW) | 0.4 | 0.3 | N/A | N/A | N/A | N/A | N/A | N/A |
| Short heel (SH) | 0.5 | 0.5 | N/A | N/A | N/A | N/A | N/A | N/A |
| Ankle circumference (AC) | 0.4 | 0.4 | N/A | N/A | N/A | N/A | N/A | N/A |
| Anatomical ball width (ABW) | 0.2 | 0.3 | N/A | N/A | N/A | N/A | N/A | N/A |

KEY: T1 = Tester 1, T2 = Tester 2. Note for Ballester [26], three devices are tested (DomeScan, Avatar 3D, and 3D scanner) so a range of values are shown here.
All values shown are in centimetres.
